# Immunosuppressive M2 TAMs represent a promising target population to enhance phagocytosis of ovarian cancer cells *in vitro*

**DOI:** 10.3389/fimmu.2023.1250258

**Published:** 2023-10-02

**Authors:** Franziska Brauneck, Leticia Oliveira-Ferrer, Jana Muschhammer, Tabea Sturmheit, Christin Ackermann, Friedrich Haag, Julian Schulze zur Wiesch, Yi Ding, Minyue Qi, Louisa Hell, Barbara Schmalfeldt, Carsten Bokemeyer, Walter Fiedler, Jasmin Wellbrock

**Affiliations:** ^1^ Department of Oncology, Hematology and Bone Marrow Transplantation with Section Pneumology, Hubertus Wald University Cancer Center, University Medical Center Hamburg-Eppendorf, Hamburg, Germany; ^2^ Mildred Scheel Cancer Career Center HaTriCS4, University Medical Center Hamburg-Eppendorf, Hamburg, Germany; ^3^ Department of Gynecology, University Medical Center Hamburg-Eppendorf, Hamburg, Germany; ^4^ 2cureX GmbH, Hamburg, Germany; ^5^ Department of Infectious Diseases, University Medical Center Hamburg-Eppendorf, Hamburg, Germany; ^6^ Institute of Hematopathology Hamburg HpH, Hamburg, Germany; ^7^ Institute of Immunology, University Medical Center Hamburg-Eppendorf, Hamburg, Germany; ^8^ Bioinformatics Core, University Medical Center Hamburg-Eppendorf, Hamburg, Germany

**Keywords:** High-grade serous ovarian cancer (HGSOC), tumor-associated macrophages (TAMs), TIGIT, repolarization, CD47, phagocytosis

## Abstract

**Introduction:**

Tumor-associated macrophages (TAMs) represent an important cell population within the tumor microenvironment, but little is known about the phenotype and function of these cells. The present study aims to characterize macrophages in high-grade serous ovarian cancer (HGSOC).

**Methods:**

Phenotype and expression of co-regulatory markers were assessed on TAMs derived from malignant ascites (MA) or peripheral blood (PB) by multiparametric flow cytometry. Samples were obtained from HGSOC patients (n=29) and healthy donors (HDs, n=16). Additional expression analysis was performed by RNAseq (n=192). Correlation with clinically relevant parameters was conducted and validated by a second patient cohort (n=517). Finally, the role of TIGIT in repolarization and phagocytosis was investigated *in vitro*.

**Results:**

Expression of the M2-associated receptors CD163, CD204, and CD206, as well as of the co-regulatory receptors TIGIT, CD226, TIM-3, and LAG-3 was significantly more frequent on macrophages in HGSOC than in HDs. CD39 and CD73 were broadly expressed on (mainly M2) macrophages, but without a clear clustering in HGSOC. CD163 mRNA levels were higher in TAMs from patients with residual tumor mass after surgery and associated with a shorter overall survival. In addition, TIGIT expression was associated with a higher tumor grading, indicating a prognostic relevance of M2 infiltration in HGSOC. TIGIT blockade significantly reduced the frequency of M2 macrophages. Moreover, combined blockade of TIGIT and CD47 significantly increased phagocytosis of ovarian cancer cells by TAMs in comparison to a single blockade of CD47.

**Conclusion:**

Combined blockade of TIGIT and CD47 represents a promising approach to enhance anti-CD47-facilitated phagocytosis.

## Introduction

High-grade serous ovarian cancer (HGSOC) is an aggressive cancer with a poor prognosis (1). It is characterized by marked tumor heterogeneity, genomic instability, and intraperitoneal spread (2, 3). The efficacy of immunotherapies remains limited (4), highlighting the unmet need to evaluate how distinct immune cell populations shape the tumor microenvironment to promote immune escape. Tumor-associated macrophages (TAMs) form an essential component of the cellular tumor microenvironment (TME). TAMs are involved in the regulation of angiogenesis, extracellular matrix remodeling, cancer cell proliferation, metastasis and immunosuppression (5). At the same time, TAMs represent a promising effector cell population as they can differentiate to develop anti-tumor functions such as tumor cell phagocytosis and antibody-dependent cellular cytotoxicity (6, 7).

The characteristic plasticity of macrophages allows polarization of resting macrophages (M0) into different states in response to different microenvironmental stimuli (8). Macrophages can be divided into two representative polarized states (within which, a variety of different activation states and functions can be further distinguished): M1 (inflammatory) and M2 (anti-inflammatory) macrophages (9). M1 macrophages are referred to as the anti-tumor effector population and are classically activated by interferon-γ (IFN-γ), tumor necrosis factor α (TNF-α), and granulocyte-macrophage colony-stimulating factor (GM-CSF). They are capable of antigen presentation, phagocytosis, cytotoxicity, and triggering T helper 1 responses (10–12). Alternative activation of immunosuppressive (M2) macrophages is induced by interleukin-4 (IL-4), interleukin-10 (IL-10) and interleukin-13 (IL-13) (13). This subpopulation can produce high levels of anti-inflammatory molecules (14). Thus, their net effect is to protect tumor cells from apoptosis while promoting angiogenesis and lymphangiogenesis as well as the immune suppression of T cells (15).

Although macrophage-based anti-tumor strategies so far have mainly focused on the inhibition of TAM recruitment (16), another novel immunotherapeutic strategy has recently shown success: the blockade of the “don’t eat me” pathway CD47/SIRPα. Blockade of this pathway restores anti-cancer phagocytic capacity (17). Increased expression of CD47 has been observed in a variety of solid tumors, including HGSOC and is associated with a worse prognosis (18, 19). Monoclonal antibodies (mAbs) that antagonize CD47 function have already shown a therapeutic potential in a number of cancers, including HGSOC (17, 20).

To develop more potent immunotherapeutic strategies based on macrophage effector function, it is necessary to gain a better understanding of the suppressive mechanisms of HGSOC-associated M2 macrophages. Moreover, we hypothesized that their anti-cancer potential may be restored by blocking immune checkpoint combinations affecting the plasticity and therefore inducing reprogramming into inflammatory effector cells. In this study, the phenotype and function of HGSOC-associated macrophages were characterized focusing on the two ends of the phenotypic spectrum: inflammatory M1 and anti-inflammatory M2 macrophages. For this purpose, macrophages from malignant ascites and peripheral blood from HGSOC patients were compared to macrophages of age-matched healthy controls. Additionally, the functional effects of blocking TIGIT on the immune profile and macrophage functions were investigated.

## Methods

### Clinical cohorts

Peripheral blood (PB) and malignant ascites (MA) aspirates were collected from patients with diagnosed HGSOC (n=26 and n=18, respectively) and compared to PB specimens from age-matched healthy donors (HD, n=16). From 13 patients we received both PB and MA. The other samples were not matched from the same patients. The RNAseq cohort includes material from 192 ovarian cancer patients who underwent primary debulking surgery according to current German guidelines (21). Cell aspirates and tumor tissue were obtained before treatment and after written informed consent in accordance with the Declaration of Helsinki and approved by the local ethics board (#200814 and PV6012-4312-BO-ff). Details of the clinical parameters are listed in **Supplementary Tables 1–3**.

### RNA extraction and transcriptome data

RNA extraction from tumor tissue of 192 ovarian cancer patients was performed as previously described (21). Briefly, tissue samples were obtained intraoperatively and immediately stored in liquid nitrogen as fresh frozen samples. The histological characteristics of each sample were evaluated on cryo-cut and hematoxylin-eosin-stained sections. If necessary, the tissue was tailored to contain at least 70% tumor. For each patient, 20–30 cryosections (approximately 16 µm) were disintegrated using precellys homogenizer (VWR International GmbH, Darmstadt, Germany) and subsequently, RNA was extracted using the RNeasy Kit (Qiagen GmbH, Hilden, Germany), according to the manufacturer’s instructions. RNA quantity and integrity were assessed using a Bioanalyzer device (Agilent, Santa Clara, CA, USA).

RNA sequencing was performed by BGI Genomics (Shenzhen, China) using the DNBseq™ Technology Platform. Sequence reads were processed with Trimmomatic (v0.38) (22) to remove sequences originating from sequencing adapters and sequences of low quality (Phred quality score below 20) and short length (below 40 bases) from the 3’ end of the sequence reads. Reads were then aligned to the human reference assembly (GRCh38.95) using STAR (v2.7.10a). Differential expression was assessed using DESeq2 (v1.34.0). A gene was considered significantly differentially expressed if the corresponding absolute log2-transformed fold change (log2FC) was not less than 1 and, in addition, the false discovery rate (FDR) did not exceed a value of 0.1.

Additionally, mRNA expression data from serous ovarian adenocarcinomas were retrieved from The Cancer Genome Atlas (TCGA) Research Network. Patient annotation data and CEL files with scans of microarrays (microarray type Affymetrix HT HG U133A) were downloaded from TCGA (The Cancer Genome Atlas Research 2011) and jointly preprocessed in Affymetrix Expression console using the robust multiarray average method (23). The characteristics of this cohort are given in **Supplementary Table 3**. The statistical analyses are described in **Supplementary Methods 2**.

### TIGIT-dependent repolarization of TAMs

TIGIT-dependent repolarization was performed on primary MA-derived TAMs. Cryopreserved mononuclear cells were isolated from the MA of patients with HGSOC (n=10). After checking the cell viability and cell count, only donors with cell viabilities above 70% were further used for the experiments. Cells were plated in 96-well (1×10^6^ cells/ml) in RPMI medium supplemented with 10% fetal bovine serum and incubated with 50 µg/mL anti-TIGIT antibody (A15153G, Biolegend) or mouse IgG2α isotype control (MG2a-53, Biolegend). After 24h, the repolarization of CD14^+^CD68^+^ cells was assessed by multiparametric flow cytometry (MFC) again using the phenotypic panels described in the **Supplementary Methods 1**. The statistical analyses are described in **Supplementary Methods 2**.

### Spheroid and tumoroid preparation protocol

During debulking surgery, solid ovarian cancer tissue samples and ascites fluid containing ovarian cancer microtumors (tumoroids n=16 and spheroids n=15) were collected from patients and processed within 24 hours. Fresh tumor fragments were frozen following cryopreservation. Subsequently, microtumors of both compartments were thawed and analyzed employing a flow cytometry panel to investigate the expression of CD47 on EpCAM^+^ CD24^+^ tumor cells in order to validate once again the expression relevance of this marker.

### Antibody-dependent phagocytosis using *in vitro* differentiated M2-like macrophages

To further test the antibody-dependent effects of the inhibitory anti-TIGIT (A15153G) and the anti-CD47 antibody (CC2C6, Biolegend), phagocytosis of ovarian cancer cells was performed *in vitro* using fresh mononuclear cells isolated from the PB of HDs. Isolated CD14^+^ monocytes were cultured and polarized into M2-like macrophages as previously described (24). M2-like cells were stained with CTred (ThermoFisher) and incubated with 50 µg/mL of the inhibitory anti-TIGIT antibody or the respective isotype control. After 24h treatment, phenotypic repolarization was evaluated by MFC. Afterward, a co-culture with CD47^+^ heat shock-induced HGSOC cell lines (OAW42, SKOV3, and OvCar3) at a 1:1 E:T ratio was started (**Supplementary Methods 3**). Tumor cells were labeled with 140 nM CellTracker™ green CMFDA (CTgreen, ThermoFisher). Heat shock was induced by warming to 47°C, over 2 hours (h). Phagocytosis was initiated by the treatment with 10 µg/ml of the inhibitory anti-CD47 antibody versus IgG1α mAb (MOPC-21, Biolegend) control. Phagocytosis was calculated as the frequency of double-positive (CTgreen and CTred) cells by MFC.

### Antibody-dependent phagocytosis using primary M2 TAMs

Primary mononuclear cells isolated from ascites fluid of patients with HGSOC (n=10) were plated into 96 well. After 24h, adherent cells were phenotypically checked for their frequency of M2 macrophages. Samples with a frequency of M2 TAMs > 30% were included in the further test. As described above cells were incubated with the blocking anti-TIGIT mAb or isotype control for 24h, followed by a co-culture with CD47^+^CTgreen^+^ apoptotic SKOV3 tumor cells and the addition of 10 µg/ml anti-CD47 antibody or isotype control for another 3h. Phagocytosis was calculated as the frequency of double positive CTgreen and CD14 cells by MFC.

## Results

### The TME of ovarian cancer contains a characteristic immunosuppressive M2 TAM subtype

Bidirectional interactions between immune and tumor cells influence the cellular tumor microenvironment to repolarize macrophages into an immunosuppressive M2 phenotype. To test whether HGSOC is associated with an increased presence of immunosuppressive macrophages, we analyzed the phenotype of primary macrophages from the ascites and PB of patients with HGSOC (n=18 and n=26, respectively) and compared them to PB-derived macrophages from healthy donors (HD n=16). Details of the multiparametric flow cytometry analyses and clinical parameters are listed in **Supplementary Methods 1** and **Supplementary Tables 1–4**.

**Figure 1:**
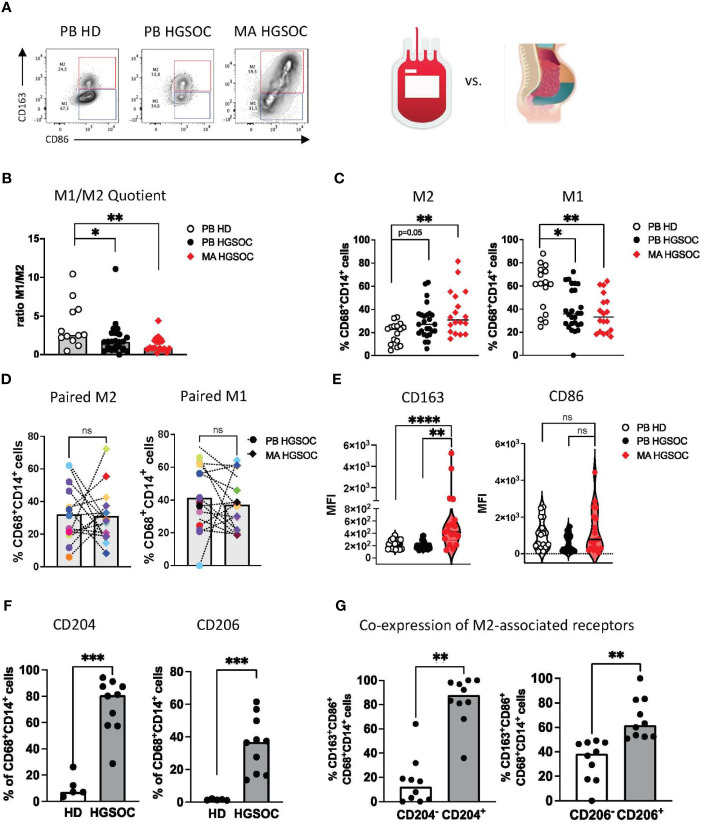
The TME of ovarian cancer contains a characteristic immunosuppressive M2 TAM subtype. Multiparametric flow cytometry (MFC) of the co-expression of CD86 and CD163 on CD14+CD68+ TAMs was performed for malignant ascites (MA) aspirates from patients with high-grade serous ovarian cancer (HGSOC n=17), for peripheral blood (PB)-derived macrophages from OvCA patients (PB n=26) and PB-derived macrophages from healthy donors (HD, n=16). **(A)** Exemplary flow plots display the M1 and M2 macrophages. **(B)** The M1:M2 ratio is depicted for each donor. **(C)** Summary data show the frequency of CD86+CD163- (M1) and CD86+CD163+ (M2) cells among CD14+CD68+ PB and MA-derived macrophages from patients with HGSOC and HDs. **(D)** Corresponding PB and MA specimens show the distribution of M1 and M2 macrophages between the two compartments. **(E)** Median fluorescence intensity (MFI) of CD163 and CD86 is depicted for PB and MA-derived macrophages. **(F)** Summary data show the frequency of CD204 and CD206 in MA-derived TAMs in comparison to PB-derived macrophages of HDs. **(G)** Summary data demonstrate the co-expression of CD163 with CD204 and CD206 on TAMs in HGSOC. Frequencies are displayed with the median. P values were obtained by the ANOVA and Kruskal-Wallis test and the Mann-Whitney test. *P<0.05, **P<0.01, ***P<0.001, ****P<0.0001, ns = statistically not significant.

Macrophages co-expressing CD68 and CD14 were differentiated into M1 (CD86^+^CD163^-^) and M2 (CD163^+^CD86^+^) phenotypes in line with published data (**Figure 1A**, **Supplementary Figure S1**) (25).

The ratio of M1:M2 macrophages was significantly lower in PB- and MA-derived TAMs from HGSOC patients than in the PB-derived macrophages from HDs (p=0.04, p=0.008, respectively, **Figure 1B**). This was caused by increased frequencies of PB- and MA-derived M2 TAMs in comparison to M2 macrophages from HDs (mean frequency PB-HGSOC vs. PB-HD: 19.7% ± 9.1 vs. 30.8% ± 15.3; p=0.05 and MA-HGSOC vs. PB-HD: 19.7% ± 9.1 vs. 35.9% ± 19.1; p=0.01), whereas PB-derived macrophages from HDs contained a higher frequency of M1 macrophages than HGSOC patients (mean frequency PB-HD vs. PB HGSOC: 58.2% ± 19.9 vs 41.12% ± 19.5; p=0.04 and PB-HD vs. MA-HGSOC: 58.2% ± 19.9 vs. 36.4% ± 16.6; p=0.0039; **Figure 1C**). Comparison of corresponding M1 and M2 TAMs derived from paired PB and MA specimens of HGSOC patients showed no difference in the distribution of the cell populations between both compartments (**Figure 1D**).

The median fluorescence intensity (MFI) analyses also showed that TAMs from the MA expressed higher levels of CD163 than TAMs from the PB (p=0.002, p<0.0001; **Figure 1E**).

Besides CD163, the scavenger receptor CD204 and the mannose receptor-1 CD206 have been described for the phenotyping of M2 TAMs. Our research confirmed this: we observed an increased frequency of CD204^+^ and CD206^+^ TAMs in the MA of patients with HGSOC in comparison to HDs (mean frequency for CD204: 73.3% ± 20.5 vs. 11.2% ± 8.9; p=0.0007 and for CD206: 35.5% ± 17.0 vs. 1.4% ± 0.4; p=0.0007; **Figure 1F**). Subsequent analyses showed that the majority of CD163^+^ TAMs co-expressed CD204 and CD206 (**Figure 1G**). In summary, TAMs derived from the PB and MA of HGSOC patients showed an increased frequency of M2-associated receptors (CD163, CD204, CD206).

### Co-regulatory molecules are more frequently expressed by M2 TAMs in ovarian cancer

We and others have recently shown that in the context of cancer, TAMs express high levels of inhibitory receptors such as TIM-3 that were originally described only on T cells (24). Therefore, we assessed the expression of the receptors TIGIT, TIM-3, CD39, and CD73 on macrophages from patients with HGSOC (PB-derived TAMs n=26, MA-derived TAMs n=18) and HDs (n=16). In addition, CD226, LAG-3 and PD-1 were analyzed for a reduced number of patients (PB-derived TAM n=21, MA-derived TAM n=15, and HDs n=12). For the gating see **Supplementary Figure S2**.

**Figure 2:**
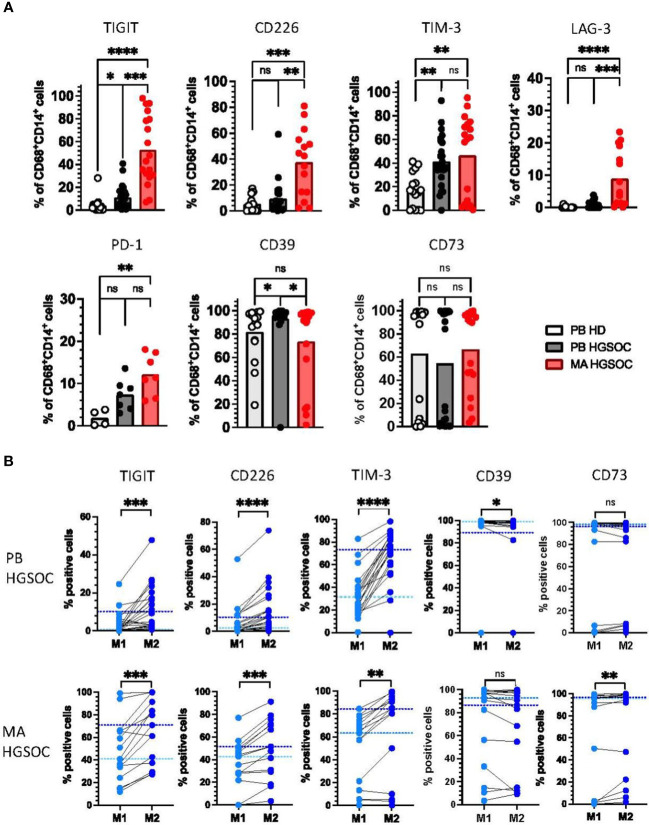
Co-regulatory molecules are more frequently expressed by M2 TAMs in ovarian cancer. Expression of the co-regulatory receptors TIGIT, CD226, TIM-3, LAG-3, PD-1, CD39, and CD73 on peripheral blood (PB)-derived CD14+CD68+ macrophages from patients with high-grade serous ovarian cancer (HGSOC n=26), malignant ascites (MA)-derived macrophages of patients with HGSOC (n=17) and PB-derived CD14+CD68+ macrophages from healthy donors (HDs n=16) was assessed by multiparametric flow cytometry. **(A)** Summary data showing the expression of the co-regulatory receptors on CD14+CD68+ macrophages. **(B)** The expression of co-regulatory receptors was compared between paired PB-derived M1 and M2 TAMs (upper panel) and MA-derived TAMs (lower panel) in HGSOC. Frequencies are displayed with the medians. P values were obtained by the ANOVA and Kruskal Wallis test and the Wilcoxon matched pairs signed rank test. *P<0.05, **P<0.01, ***P<0.001, ****P<0.0001, ns = statistically not significant.

In comparison to HDs, PB- and MA-derived TAMs from HGSOC patients displayed higher frequencies of TIGIT (p=0.028, p=0.0001) and TIM-3 (p=0.0074, p=0.0035), whereas LAG-3, PD-1 and the costimulatory receptor CD226 were only more frequently expressed by MA-derived TAMs (p<0.0001, p<0.01 and p=0.0003; **Figure 2A**).

Interestingly, the ectoenzyme CD39 was higher expressed by PB-derived, but not MA-derived macrophages in comparison to HDs (p=0.02; **Figure 2A**). The frequency of CD73^+^CD68^+^CD14^+^ cells was similar in all three patient cohorts (**Figure 2A**). Of note, donors in all three groups had either a very high or a very low expression of CD73, while there were hardly any donors with a medium CD73 expression.

Because M2 TAMs have been described as immunosuppressive, we hypothesized that expression of the inhibitory receptors would be higher on the M2 than on the corresponding M1 TAMs. Comparative analyses were performed on PB- and MA-derived TAMs in HGSOC (n=21, n=15). In both tissue compartments, the TIGIT, CD226, TIM-3, LAG-3 and PD-1 expressing TAMs were more frequent in the M2 than in the M1 population (**Figure 2B**, **Supplemental Figure 3A**). By contrast, CD39^+^ cells were less frequent among M2 TAMs, however this was only observed for PB-derived macrophages. No differences in expression were detected for CD73 between both TAM subtypes (**Figure 2B**).

Collectively, these data indicate that in PB- and MA-derived macrophages from high-grade ovarian cancer patients the increased frequency of TIGIT, CD226, TIM-3, and LAG-3 positive cells is associated with an M2 phenotype.

### CD226, TIM-3 and LAG-3 are co-expressed with TIGIT by (M2) TAMs

**Figure 3:**
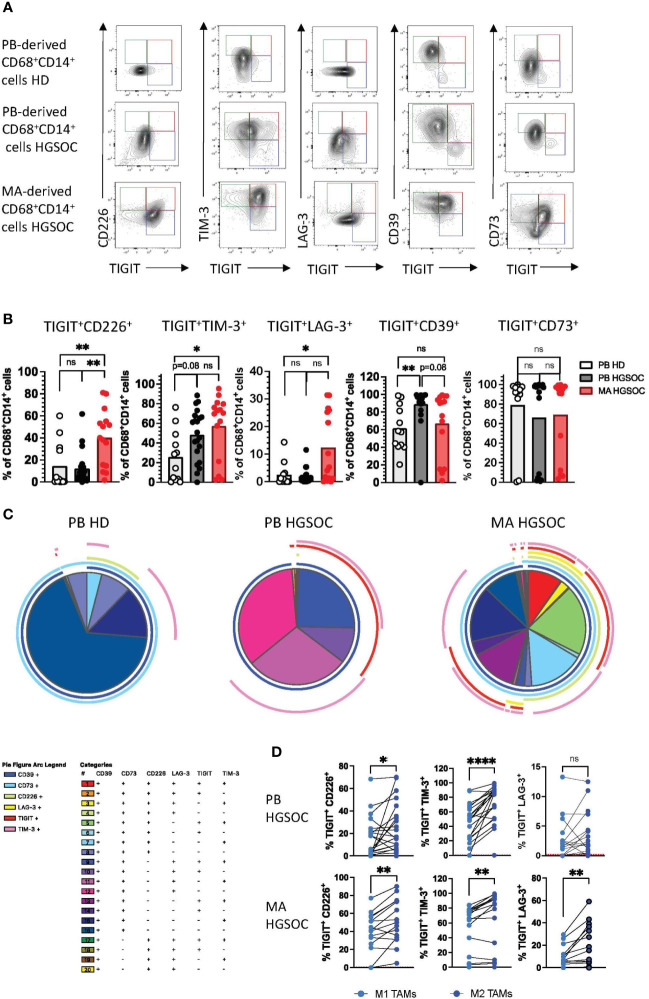
CD226, TIM-3 and LAG-3 are co-expressed with TIGIT by (M2) TAMs. Co-expression patterns of the co-regulatory receptors by CD14+CD68+ macrophages were analyzed for peripheral blood (PB)-derived macrophages from healthy donors (HDs n=16) or high-grade serous ovarian cancer patients (HGSOC n=26), as well as for malignant ascites (MA)-derived macrophages from HGSOC patients (n=17). **(A)** Exemplary flow plots display characteristic co-expression patterns of co-regulatory molecules. **(B)** Summary data showing the frequency of macrophages co-expressing the indicated co-regulatory receptors. **(C)** Spice analyses showing the pattern of co-expression of co-regulatory receptors by PB and MA-derived macrophages. **(D)** Co-expression of any two of the co-inhibitory receptors TIGIT, TIM-3, and LAG3 is compared between paired M1 and M2 PB- derived TAMs in HGSOC (upper panel) and MA-derived TAMs (lower panel). P values were obtained by the ANOVA and Kruskal-Wallis test and by the Wilcoxon matched-pairs signed rank test. *P<0.05, **P<0.01, ****P<0.0001, ns = statistically not significant.

Co-expression of multiple inhibitory receptors has been described as an important feature of T-cell exhaustion (26). Based on the high frequencies of TAMs expressing TIGIT, CD226, TIM-3, LAG-3, PD-1, CD39, and CD73, we next analyzed the co-expression of these receptors (PB-derived TAM n=21, MA-derived TAM n=15 and HDs n=12). TIGIT^+^ macrophages accounted for the largest proportion of cells co-expressing further co-regulatory receptors (**Figure 3A**). In detail, our analyses showed that in comparison to HDs, MA-derived TAMs contained elevated frequencies of TIGIT^+^CD226^+^ (p=0.001), TIGIT^+^TIM-3^+^ (p=0.01), TIGIT^+^LAG-3^+^ (p=0.03) and TIGIT^+^PD-1^+^ (p<0.05) cells (**Figure 3B**, **Supplementary Figures 3B, C**). PB-derived macrophages from HGSOC patients revealed only higher percentages of TIGIT^+^TIM-3^+^ (p=0.08) and TIGIT^+^CD39^+^ (p=0.0056) cells versus HDs (**Figure 3B**). As illustrated in the Simplified Presentation of Incredibly Complex Evaluations (SPICE) analysis, macrophages isolated from the ascites displayed the highest proportion of cells co-expressing the regulatory receptors (**Figure 3C**). Next, we compared the co-expression patterns between M2 and M1 macrophages. M2 TAMs showed a higher co-expression of TIGIT with TIM-3, LAG-3, and CD226 in comparison to their corresponding M1 TAMs (**Figure 3D**). In summary, co-expression of inhibitory receptors is a hallmark of the M2 phenotype in ovarian cancer, with the highest frequencies of co-expressing cells being found within the MA compartment.

### High expression of CD163^+^ confers a negative prognosis in ovarian cancer patients

**Figure 4:**
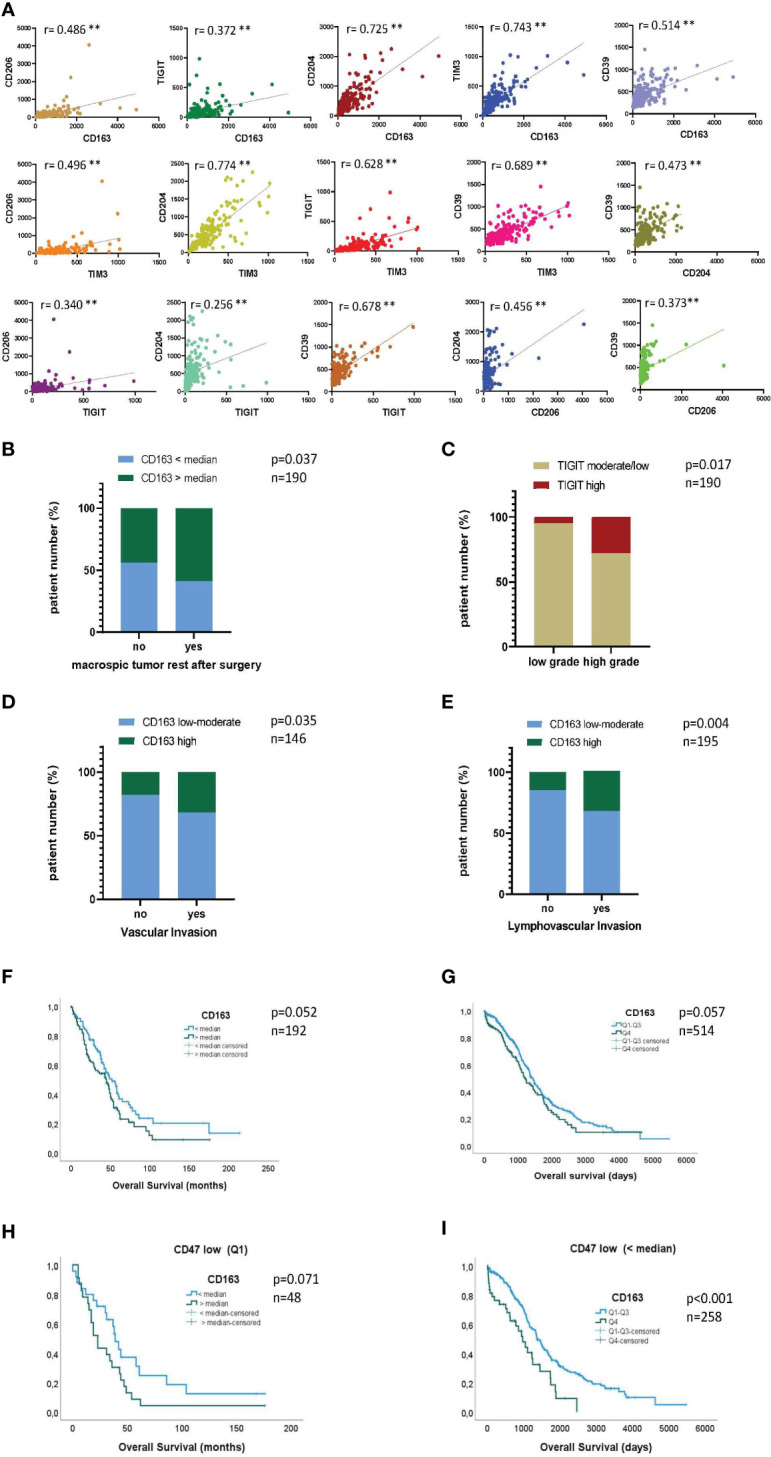
High expression of CD163 confers a negative prognosis in ovarian cancer patients. Gene expression of CD163, CD204, CD206, TIGIT, TIM-3 and CD39 was assessed in a cohort of 192 patients diagnosed with high-grade serous ovarian cancer (HGSOC): Correlation analyses between **(A)** normalized expression values of the indicated markers, **(B)** CD163 levels and the presence of residual tumor mass after resection and **(C)** TIGIT levels and tumor grade. (F+H) Kaplan Meier analyses showing overall survival (OS) of patients with high (gene expression > the median) or low (gene expression < the median) CD163 gene expression for the entire patient cohort **(F)** and stratified for CD47 mRNA expression **(H)**. In addition, correlation of CD163 gene expression was analyzed with clinical parameters of the TCGA cohort (n=517): (D+E) Correlation between CD163 levels and vascular **(D)** or lymphovascular **(E)** invasion status is depicted. (G+I) Kaplan Meier analyses showing overall survival (OS) of patients with high or low CD163 gene expression for the entire cohort **(G)** and stratified for the expression of CD47 **(I)**. P values were obtained by Chi-square and log-rank tests. **P<0.01.

Next, we aimed to relate the immunoprofiling results to molecular gene expression data. We therefore analyzed the correlation between gene expression of the most dominant M2 receptor CD163 and the additional M2-associated receptors CD204 and CD206. In addition, we investigated the correlations between the inhibitory checkpoint receptors TIGIT, CD39, and TIM-3 and the aforementioned M2 receptors at the gene expression level. Therefore, analysis of tumor tissue samples from HGSOC patients (n=192) were conducted using available RNAseq data from the gynecology department at the UKE. Details of the cohort and clinical parameters are listed in **Supplementary Table 2**. CD163 expression levels positively correlated with those of CD204 and CD206 (r=0.486, p<0.001 and r=0.725, p<0.001; **Figure 4A**). In addition, high levels of TIGIT, TIM-3 as well as CD39 significantly correlated with high CD163, (but also CD204, and CD206) expression. Moreover, expression of TIGIT was also associated with TIM-3 and CD39 expression (**Figure 4A**). The results are in line with our phenotypic data from protein analysis in PB and MA, in which increased frequencies of TIGIT^+^ and TIM-3^+^ cells were detected in the M2 TAM population, with a significant proportion of cells co-expressing these molecules. In contrast to the RNA data, protein analyses did not show significantly increased expression of CD39 on M2 macrophages. Next, the expression levels of the most relevant markers in this study, namely CD163 and TIGIT, were related to available clinical-histopathological parameters. Interestingly, CD163 mRNA levels were significantly higher in tumors from patients with residual tumor mass after surgery (**Figure 4B**). Accordingly, high CD163 levels were strongly associated with shorter overall survival (**Figure 4F**; p=0.052). Moreover, when we stratified the cohort into CD47 high vs. low expressors, only in the group of CD47 low expressors showed a trend towards the negative impact of high CD163 expression on overall survival was observed (**Figure 4H**, **Supplementary Figure 4A**; p=0.071 and p=0.687). This observation may indicate that CD47-low-expressing tumor cells are more resistant to macrophage surveillance and that blocking CD47 in low-expressing cells may not be sufficient for effective tumor control. Therefore, the combination with anti-TIGIT could improve phagocytosis especially in this subgroup of patients. TIGIT mRNA levels were significantly higher in high grade tumors (**Figure 4C**). For TIM-3, CD204, or CD206 no significant correlation with these clinical parameters was found. Additionally, using publicly available microarray data (TCGA; cohort description **Supplementary Table 3**) we could validate the strong and significant correlation between CD163 and CD206, (r=0.519, p<0.001) as well as between CD163 and CD39 (r=0.537, p<0.001) (**Supplementary Table 5**). Further, we corroborated the strong association between high CD163 mRNA levels and poor patient prognosis (n=514, p=0.057; **Figure 4G**). Again, when stratifying for CD47, the negative impact of high CD163 mRNA levels were only observed in the CD47 low expression group (**Figure 4I**, **Supplementary Figure 4B**; p<0.001 and p=0.687). Furthermore, a correlation with vascular (n=146, p=0.035; **Figure 4D**) and lymphovascular invasion (n=195, p=0.004; **Figure 4E**) and high CD163 mRNA levels was found, both features indicative of an aggressive and invasive tumor phenotype. TIGIT and TIM-3 levels were not available in the TCGA cohort and therefore analysis including these factors could not be performed. In summary, our data show that the expression of M2-associated receptors correlates with each other, as well as with the expression of inhibitory molecules. Furthermore, the expression of CD163 and TIGIT was associated with unfavorable clinical parameters, indicating a prognostic relevance of M2 infiltration in ovarian cancer.

### Blockade of TIGIT induces phenotypic repolarization of macrophages *in vitro*

**Figure 5:**
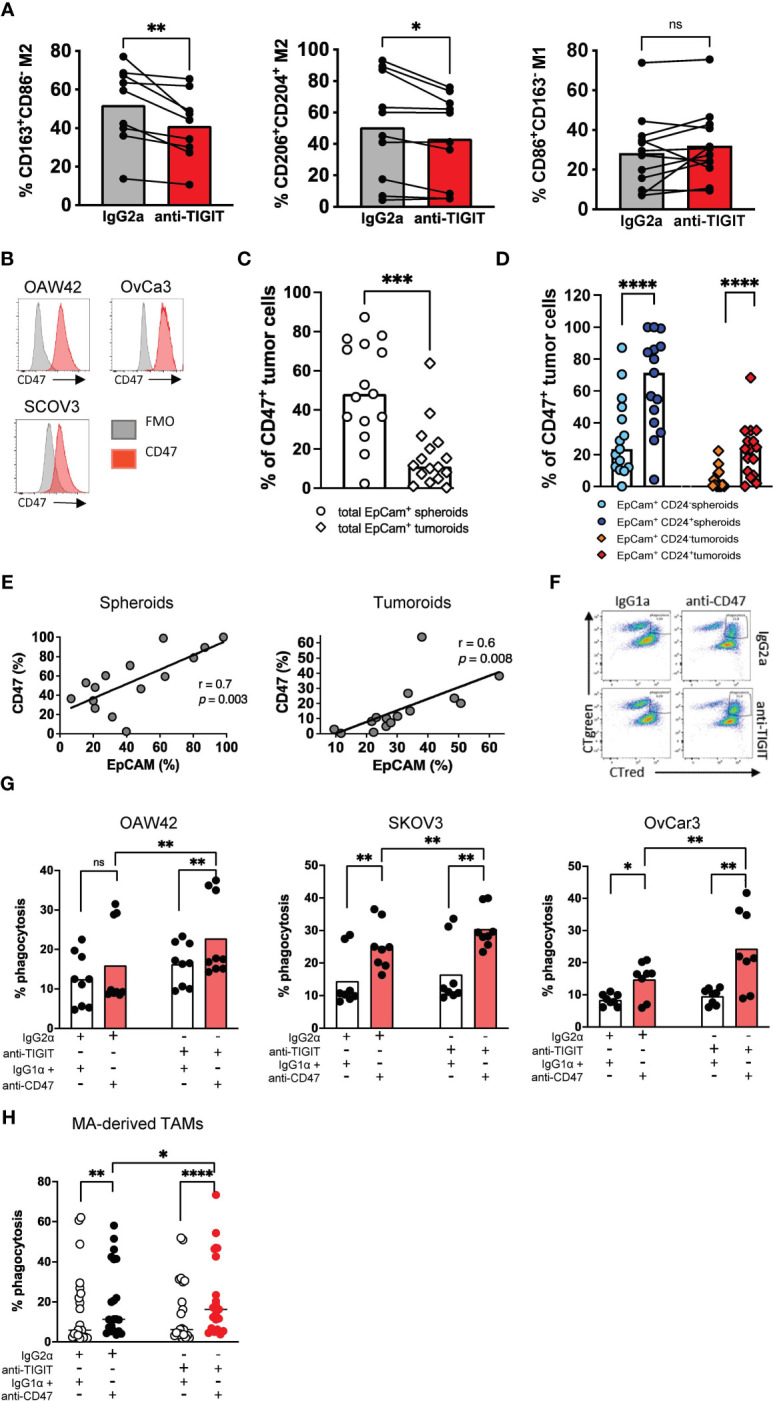
Blockade of TIGIT induces a phenotypic repolarization and increases CD47-dependent cellular phagocytosis *in vitro*. Blockade of TIGIT induces the repolarization of M2 macrophages *in vitro*. **(A)** Primary malignant ascites (MA)-derived macrophages from high-grade serous ovarian cancer (HGSOC) patients (n=7) were incubated with anti-TIGIT or control antibodies. Expression of CD163, CD206, CD204 and CD86 was assessed after 24h. Measurements were performed in technical triplicates. **(B)** Flow histograms display the expression of CD47 (red histogram) on ovarian cancer cell lines in comparison with the fluorescence minus one (FMO) control (gray histogram). **(C)** Summary data show the expression of CD47 on EpCAM+ spheroids and tumoroids. **(D)** CD47 expression is compared between different spheroid and tumoroid subpopulations. **(E)** The frequency of CD47+ cells was correlated with that of EpCAM+ spheroid and tumoroid cells. **(F)** Representative flow plots showing phagocytosis of ovarian cancer cell lines (labelled green) by peripheral blood (PB)-derived macrophages (labelled red). Phagocytosis was assessed as described in the Material and Methods section. Phagocytosis events appear as double-positive cells in the right upper quadrant. **(G)** Antibody-mediated phagocytosis of OvCA cell lines OAW42 (n=3), SKOV3 (n=3) and OvCar3 (n=3) by PB-derived macrophages was analyzed as described above. **(H)** Antibody-mediated phagocytosis of SKOV3 cells by primary MA-derived TAMs is depicted following single or combined blockade of TIGIT and CD47. Measurements were performed in technical triplicates. P values were obtained by the Wilcoxon matched-pairs and the ANOVA and Friedman signed rank test. *P<0.05, **P<0.01, ***P<0.001, ****P<0.0001, ns = statistically not significant.

Due to the high TIGIT expression on M2 macrophages, we set out to analyze the therapeutic potential of blocking TIGIT. In previous studies, we investigated the effects of a TIGIT blockade on the polarization of PB monocyte-derived macrophages *in vitro.* TIGIT blockade decreased the frequency of M2-like macrophages and increased the proportion of M1-like macrophages (24). The present study aimed to investigate this effect on primary TAMs. Mononuclear cells from the MA of 10 HGSOC patients were cultured in the presence of an anti-TIGIT antibody or its respective isotype control mAb for 24h. Afterward, the M2 (CD163^+^CD86^+^) and M1- (CD163^-^CD86^+^) phenotype was re-assessed by MFC (**Supplementary Figure 5A**). Treatment with the anti-TIGIT antibody did not affect the viability or the proportion of CD68^+^CD14^+^ TAMs in the total cell culture, indicating that anti-TIGIT treatment had no cytotoxic effect on TAMs (**Supplementary Figures 5B, C**). While TIGIT blockade did not affect the percentage of cells expressing the M1 phenotype, it significantly reduced the frequency of macrophages expressing the M2-associated receptors including CD163, CD204 and CD206 (**Figure 5A**, **Supplementary Figure 5D**). In conclusion, 24h blockade of TIGIT resulted in a phenotypic shift by reducing the frequency of primary M2 TAMs.

### Blockade of TIGIT increases CD47-dependent cellular phagocytosis *in vitro*

It was recently reported that CD47 expression is upregulated in ovarian cancer cells and associated with poor prognosis and the infiltration of CD163^+^ TAMs (18, 19). The OAW42, SKOV3, and OvCar3 ovarian cancer cells highly express CD47 (**Figure 5B**). Moreover, CD47 expression was also analyzed on primary spheroids derived from the MA (n=16) and tumoroids derived from primary tumor tissue (n=15) of ovarian cancer patients. The frequency of CD47^+^ tumor cells was significantly higher in spheroids from MA than in tumoroids from tumor tissue (**Figure 5C**). In addition, spheroids and tumoroids were further differentiated regarding their co-expression of EpCAM and CD24. CD24, a tumor stem cell marker in ovarian cancer, is involved in the regulation of the epithelial-mesenchymal transition and is a predictor of the clinical outcome. The frequency of CD47^+^ cells was higher within the double positive (EpCAM^+^CD24^+^) tumor cell population than in the EpCAM^+^CD24^-^ tumor cell fraction in both the spheroids and the tumoroids (**Figure 5D**). The frequency of CD47^+^ cells positively correlated with the frequency of EpCAM^+^ tumor cells within both spheroids and tumoroids (**Figure 5E**).

Based on the high frequency of CD47^+^ ovarian cells and TIGIT^+^ M2 TAMs, we hypothesized that the blockade of TIGIT could stimulate CD47-mediated phagocytosis by repolarizing M2 into a more inflammatory M1 state. Therefore, we investigated whether the combined blockade of TIGIT and CD47 could augment phagocytosis compared to CD47 monotherapy.

Monocyte-derived macrophages from HDs (n=3) were differentiated into M2-like macrophages over 6 days *in vitro* and treated with an anti-TIGIT antibody or isotype control mAb for 24h. After evaluating the successful repolarization into M1 macrophages, the macrophages were co-cultured for 4h with CD47^+^ ovarian cancer cell lines in the presence of an anti-CD47 mAb or isotype control mAb. We observed a significantly increased phagocytosis by the sequential blockade of the TIGIT and CD47 receptors for OAW42 (n=3), SKOV3 (n=3), and OvCar3 (n=3) cells in comparison to single blockade of CD47 as measured by MFC (mean phagocytosis (%) of OAW42: combined blockade vs. CD47 blockade 22.8 ± 10.2 vs. 16.0 ± 10.4, p=0.0039; for SKOV3: combined blockade vs. single blockade 30.5 ± 6.1 vs. 25.1 ± 7.2, p=0.0078; and for OvCar3: combined blockade vs. single blockade 23.6 ± 12.2 vs. 14.8 ± 5.5, p=0.0078; (**Figures 5F, G**).

Next, we assessed the effect on primary patients’ samples. Mononuclear cells were obtained from the MA of 7 patients diagnosed with HGSOC. After 24h of treatment with either anti-TIGIT or the isotype control mAb, incubation with anti-CD47 or its isotype control was sequentially performed for additional 4h. Again, the combined blockade of TIGIT and CD47 significantly increased phagocytosis of SKOV3 cells by primary MA-derived TAMs (defined by double positive CTgreen and CTred cells) in comparison to a blockade of CD47 alone or the isotype control (mean phagocytosis (%) of combined blockade vs. CD47 blockade 23.6 ± 20.1 vs. 20.1 ± 18.1, p=0.01 **Figure 5H**). Taken together, the blockade of TIGIT induced a phenotypic repolarization defined by a reduction of M2-associated receptors and ultimately resulted in a significant augmentation of the anti-CD47-mediated ovarian cancer phagocytosis.

## Discussion

The present study showed that the PB and MA of patients with HGSOC contain an increased frequency of immune suppressive TAMs expressing M2 markers such as CD163, CD204 and CD206, and a decreased frequency of M1 macrophages. In addition, HGSOC-derived M2 TAMs showed an increased expression of the co-inhibitory receptors TIGIT, TIM-3 and LAG-3, whereas CD39 was more frequently expressed by M1 TAMs. Our expression data revealed characteristic co-expression patterns, such as the significantly clustered co-expression of TIGIT with CD226, TIM-3, and LAG-3. To investigate the prognostic relevance of the above markers for a larger cohort of patients, we analyzed RNAseq data from 192 HGSOC patients. The expression of the dominant M2 marker CD163 was correlated with the expression of other M2 markers such as CD204 and CD206. In addition, the expression of CD163 was associated with a higher expression of TIGIT and TIM-3. Expression of CD163 and TIGIT was significantly higher in patients with a more aggressive and invasive tumor subtype. Where the increased CD163 expression was also associated with a shorter OS. Performing a monoclonal antibody blockade of TIGIT, the phenotypic repolarization of TIGIT^+^ M2 TAMs into M1 macrophages was induced in an *in vitro* model. This repolarization could also be shown on a functional level, since TIGIT blockade significantly augmented the phagocytic capacity of macrophages in combination with blockade of CD47.

A unique property of macrophages is their high plasticity (8). This allows them to switch back and forth between different subtypes, which is controlled by intrinsic and extrinsic factors (27). Especially for TAMs, different phenotypes could be found depending on the external stimuli of the tumor microenvironment (27, 28). One of the most important external regulators are the tumor cells themselves as they can secrete M2-like cytokines such as IL-10, CCL2/3/4/5, CXCL12, VEGF, and platelet-derived growth factor (PDGF) to recruit monocytes and M0 macrophages to the tumor site and differentiate them into suppressive M2 TAMs (29, 30). Through this process, the majority of intra-tumoral macrophages exhibit an M2 phenotype, which correlates with a poor prognosis in a number of malignancies (27, 31). We recently showed that a significantly altered M2:M1 ratio was also present in hematological malignancies: In the bone marrow of patients with acute myeloid leukemia (AML), the frequency of M2 macrophages was significantly increased in patients with active AML (initial diagnosis and relapse) in comparison to healthy donors or AML patients in remission (24). The present study similarly demonstrated that the M1/M2 ratio was reversed in the PB and MA of patients with HGSOC in comparison to HDs. M2 macrophages represented the dominant subpopulation in HGSOC patients PB and MA. CD163 was mainly used since CD204 and CD206, two other M2-associated receptors, were largely co-expressed on CD163 TAMs, to define M2 macrophages in this study. Thus, we limited phenotyping of macrophages to CD163, CD86, CD68 and CD14 for simplicity (25, 32, 33).

*TIGIT, TIM-3, LAG-3 and PD-1* were initially identified as membrane markers specific for T-lymphocytes, but their expression was recently found on further immune cells (34, 35). The present study reveals their expression on TAMs, particularly on M2 TAMs. Moreover, we observed a characteristic co-expression pattern of TIGIT with TIM-3 and LAG-3. The TIGIT/PVR and the TIM-3 pathways have recently been shown to be involved in the macrophage polarization, suppression of pro-inflammatory cytokine production, and cancer cell growth in several mouse models (36–38). In line with our data, the three inhibitory receptors represent promising M2 targets whose blockade, and the macrophage effects mediated thereby should be further investigated.

In contrast to the inhibitory receptors, CD226 plays a distinct role as a co-stimulatory receptor. Interestingly, we found a higher frequency of CD226^+^ TAMs and significant co-expression of CD226 and TIGIT on HGSOC-derived M2 TAMs. Beyer et al. identified CD226 also as a novel M2-associated cell surface marker (39). Its co-expression with TIGIT could be due to the fact that CD226 is involved in early macrophage migration. CD226-deficient macrophages/monocytes can no longer migrate into the tissue (40). In addition, it has been reported that CD226 on macrophages plays a role in antigen presentation (41). In previous studies, we could also show that CD226 expression was upregulated after TIGIT blockade on macrophages (24).

CD39 was expressed by the majority of macrophages. Under inflammatory stress, Cohen et al. demonstrated that Toll-like receptor (TLR)-stimulated macrophages modulate their activation state by increased production of adenosine triphosphate (ATP). Subsequent degradation into adenosine by CD39 generates an immunosuppressive environment (42). Increased extracellular ATP levels were associated with increased phagocytosis and dysregulated chemotaxis of macrophages (43). Blockade of CD39, which is expected to increase local extracellular ATP concentrations, enhanced macrophage secretion of TNF-α and IL-12 and reduced IL-10 release (44). These data suggest that CD39 plays a critical role in the self-limitation of inflammatory processes, particularly by M1 macrophages. This might be a mechanism to diminish the inflammatory activities of M1 macrophages in situations with chronic immune stimulation, such as cancer and autoimmune diseases. Additionally, the study revealed no difference in the frequency of *CD73* ^+^ macrophages between healthy donors and HGSOC patients. However, compared with the expression of CD39 in general, we found a significantly lower frequency of positive cells. Interestingly, however, a clear pattern emerged in that donors showed either a high or a low frequency of CD73^+^ cells. It should be further investigated if there is a strong genetic component driving the CD73 expression, which may determine interindividual differences.

Progression of cancer is often associated with an increased dysfunction of T cells. In addition, recent studies also described dysfunction in further cell populations including innate cells e.g. NK cells. One hallmark of immunologic dysfunction is the aberrant co-expression of multiple inhibitory receptors. Interestingly, this study detected a characteristic cluster of multiple inhibitory receptors co-expressed on immunosuppressive M2 macrophages. Whether the co-expression of multiple inhibitory receptors represents also a characteristic state of progressive macrophage exhaustion remains to be investigated.

The RNAseq data from the UKE ovarian cancer patient cohort showed that the expression of CD163 was correlated with other M2 markers, such as CD206 and CD204, and was also positively associated with the expression of TIGIT and TIM-3. These data could largely be confirmed in a second patient collective (TCGA cohort) and support the findings of our phenotypic studies. With regard to clinical parameters, we found an association between high CD163 expression and shorter survival, as well as between high CD163 and TIGIT levels and features of a more aggressive and invasive tumor growth, such as residual tumor after surgery, vascular and lymphovascular invasion in HGSOC patients. An unfavorable impact of CD163 expression on overall survival has also been observed for other tumor entities (45). Similar to our findings, a retrospective study analyzing patients with stage III-IV HGSOC that used CD163 as an M2 marker found a significant difference in overall as well as progression-free survival between high and low CD163 expression (46). Multivariate analysis in this study identified the ratio of CD163/CD68 (essentially mirroring the M2:M1 ratio) as a negative prognostic predictor. In addition, a meta-analysis of 9 studies that included 794 HGSOC patients validated a high M1/M2 ratio in tumors as a relevant marker for improved prognosis (47).

Therapeutically this study showed that TIGIT^+^ M2 macrophages (both, PB-derived M2 macrophages from HDs as well as M2 TAMs from HGSOC patients) can be repolarized towards an M1 phenotype upon antibody-based TIGIT blockade *in vitro*. These data demonstrate that the immunosuppressive plasticity of TAMs is directly regulated by immune checkpoints. We recently found similar repolarization effects following TIGIT blockade on AML-derived macrophages. In this recently published study, we also detected an effect of blocking TIGIT on the cytokine production of macrophages. Blockade of TIGIT resulted in increased secretion of proinflammatory TNF-α, IL-1β, IL-1RA, CXCL-10, and CCL-17 by the macrophages suggesting that blockade of TIGIT not only repolarizes the phenotype and thus increases anti-cancer phagocytosis but also enhances the cytotoxicity via increased secretion of inflammatory cytokines and chemokines in tumor-associated macrophages (24). We found no further comparable studies for the blockade of TIGIT on macrophages in the literature. However, in a mouse model, binding of TIGIT to CD155 on M1 macrophages induced polarization to IL-10-secreting M2 macrophages (36). These data suggest that the TIGIT/CD155 pathway has an important role not only in T cell function but also in macrophage polarization.

Finally, sequential blockade of TIGIT and CD47 resulted in increased phagocytosis of HGSOC cells *in vitro*. The combined blockade enhanced phagocytosis of HGSOC cell lines by *in vitro* differentiated PB-derived as well as primary MA-derived M2 macrophages. We have also recently described this effect for TIGIT^+^ AML-derived macrophages (24). Based on these results, we assume that this immunological mechanism can be applied to different types of tumors. Since HGSOC tumor cells express high levels of the “don´t eat me” ligand CD47, and high CD47 expression is associated with a worse prognosis in metastatic ovarian tumors (18), CD47 represents a promising target to increase antibody-mediated phagocytosis through diminished “don´t eat me” signals. The focus of our experimental setting was to measure the levels of phagocytosis. The anti-CD47 clone CC2C6 which we used for our experiments, was shown to induce phagocytosis by disrupting the interacting between SIRP1 and CD47 (48). However, this antibody clone was also described to induce antibody-dependent cellular cytotoxicity (ADCC) (49). Both mechanisms, the disruption of the “don’t eat me” signal and a signal mediated by the FcR of the CD47 antibody, might induce the phagocytosis. Complementary to the CD47 effect, the blockade of TIGIT is based on a second immunological mechanism, the repolarization of macrophages. As demonstrated in our studies, the combined blockade of TIGIT and CD47 could further enhance the potential of macrophage-mediated phagocytosis by upstream repolarization of M2 into M1 macrophages. Blocking TIGIT might also represent a beneficial combination with other phagocytosis inducing antibodies, either via blocking other “don’t eat me” signals or via other targets inducing FcR-mediated phagocytosis. However, this issue was beyond the scope of our current work and should be investigated in further studies.

In conclusion, the combined blockade of TIGIT and CD47 represents a promising approach to further enhance anti-CD47-mediated phagocytosis. The findings of our study can directly be translated into clinical trials since studies with anti-CD47 and anti-TIGIT antibodies are ongoing and respective antibodies are clinically available.

## Data availability statement

The complete RNA sequencing datasets presented in this article are not readily available due to data protection policies. Requests to access the datasets should be directed to Leticia Oliveira-Ferrer (ferrer@uke.de).

## Ethics statement

The studies involving humans were approved by Ethikkommission der Ärztekammer Hamburg, Weidestr. 122 b, 22083 Hamburg, Germany. The studies were conducted in accordance with the local legislation and institutional requirements. The participants provided their written informed consent to participate in this study.

## Author contributions

FB, LO-F, WF, and JW designed the research study, performed the experiments, analyzed the data, and wrote the manuscript. JM, YD, MQ, and LH performed the experiments and analyzed the data. TS and CA analyzed the data and reviewed the manuscript. JS and FH oversaw the interpretation and reviewed the manuscript. BS and CB provided samples and material and reviewed the manuscript. All authors contributed to the article and approved the submitted version.
