# Supplementary material for: Immunosuppressive M2 TAMs represent a promising target population to enhance phagocytosis of ovarian cancer cells in vitro
Source: Front Immunol. 2023 Oct 2;14:1250258. doi: 10.3389/fimmu.2023.1250258 (PMC10593434; doi:10.3389/fimmu.2023.1250258)
Supplement: Supplementary file 1 [file DataSheet_1.pdf]

Supplementary Material

Immunosuppressive M2 TAMs represent a promising target population to enhance phagocytosis of ovarian cancer cells in vitro

Franziska Brauneck[1,2], Leticia Oliveira-Ferrer[3], Jana Muschhammer[1], Tabea Sturmheit[4], Christin Ackermann[5], Friedrich Haag[6], Julian Schulze zur Wiesch[5], Yi Ding[3], Minyue Qi[7], Louisa Hell[3], Barbara Schmalfeldt[3], Carsten Bokemeyer[1], Walter Fiedler[1] and Jasmin Wellbrock[1]

*** Correspondence:** Franziska Brauneck: f.brauneck@uke.de

1 Supplementary Methods

Supplementary Methods 1: Multiparametric Flow Cytometry (MFC).
Cryopreserved cells were stained for multiparametric flow cytometry as previously described [28]. In brief, mononuclear cells were washed, FCR block (FcR blocking reagent, human, Miltenyi Biotec) was added, and cells were stained with LIVE/DEAD fixable Near-IR dye (Thermo Fisher) according to the manufacturer's protocol to exclude dead cells. Surface proteins were stained after a second FCR blockade by incubation with appropriate fluorochrome-conjugated antibodies (the panels used are shown in their entirety in **Supplementary Table 2**) for 20 min at RT in the dark.
For intracellular staining, the cells were permeabilized for 40 min using the FOXP3 Fixation/Permeabilization Buffer Set (eBioscience) according to the manufacturer's instructions. Thereafter, cells were intracellularly stained with the fluorochrome-labeled intracellular antibody and incubated for 30 min at 4°C in the dark.
The gating strategy is demonstrated in **Supplementary Figure S1**. Compensation controls were measured using single-stained Comp Beads (Anti-Mouse Ig,κ/Negative Control Compensation Particles Set, BD Biosciences). All samples were run on a BD FACSymphony A3 with FACS Diva version 8 (BD Biosciences).

Supplementary Methods 2: Statistical Analyses.
Flow cytometry data were analyzed using the FlowJo 10.5.2. software (Treestar) as previously described [28]. Statistical analysis was carried out using Prism 9.0 software (GraphPad Software). A comparison of individual unpaired samples was carried out using the Mann-Whitney test. For paired analyses, we used the Wilcoxon matched-paired signed-rank test. The Kruskal-Wallis or Friedmann test were used for more than two groups, respectively. Pearson's correlation and Spearman's rank correlation coefficient were applied for bivariate correlation analysis. Frequencies in the text are described as medians unless stated otherwise. Display of multicomponent distributions was performed with SPICE v5.1. For the statistical analysis of clinical data, we used SPSS Statistics version 27 for Windows (IBM, Armonk, NY, USA). Correlations between mRNA levels were assessed using two-sided Pearson tests. Chi-square tests were used to correlate mRNA levels with clinical and histopathological parameters such as stage, FIGO, grading, nodal involvement and residual tumor after surgery. Kaplan-Meier estimates and the log-rank test were carried out to ascertain and compare disease-free and overall survival. P-values smaller than 0.05 were considered significant, where *, **, and *** indicate p-values below 0.05, below 0.01, and 0.001, respectively.

Supplementary Methods 3: Ovarian cancer cell lines.

OAW42 is a human ovarian adenocarcinoma cell line, established from the ascites of a patient with ovarian cystadenocarcinoma. The human SKOV3 cell line is also an ovarian adenocarcinoma cell line but was isolated from the ovary. OvCar3 was isolated from the malignant ascites of a high-grade serous adenocarcinoma patient, refractory to cisplatin.

All three cell lines were purchased from the ATCC (American Type Culture Collection, Germany). OAW42 cells were cultured in Dulbecco's Modified Eagle's Medium (DMEM, ThermoFisher) supplemented with 10% fetal bovine serum (FBS, Gibco, Thermo Fisher Scientific). SKOV3 cells were cultured in Mc Coy’s modified 5A medium (ThermoFisher) supplemented with 10% FBS and OvCar3 cells were cultured in Roswell Park Memorial Institute (RPMI) 1640 Medium (ThermoFisher) supplemented with 10% FBS.

All three ovarian cancer cell lines express CD47, which was regularly evaluated by flow cytometry.

2 Supplementary Tables

Supplementary Table 1. OvCA Gyn UKE cohort (AG Oliveira Ferrer, RNAseq).

Patient number	Diagnosis	Histology	Grading	FIGO	Nodal involvement	M	pT	Residual Tumor	Primary/ recurrent	Material
#1	HGSOC	mucinous	hg	IIIa1	pN1b	0	pT1a		primary	PB
#2	MMMT		hg	IV	pN1a	1	pT3b	macroscopic tumor free	primary	PB / MA
#3	HGSOC	serous	hg	IIIc	pN1a	0	pT2a	> 1cm	primary	MA
#4	MMMT		hg	IIIa	pN1b	0	pT2b	macroscopic tumor free	primary	PB
#5	HGSOC	serous	hg	IIIc		0	pT3c	< 1cm	remission /neoadjuvant	PB
#6	HGSOC	endometrioid	lg	IIIc	pN0	0	pT3c	macroscopic tumor free	primary	PB
#7	HGSOC	serous	hg	IIIc	pN1	0	pT3C	macroscopic tumor free	recurrent	PB / MA
#8	HGSOC	serous	hg	IVb	pN1a	1	pT3c	> 1cm	primary	PB / MA
#9	HGSOC	serous	hg	IIIc	ypN1a	0	ypT3c	1cm	primary/neoadjuvant	PB / MA
#10	HGSOC	serous	hg	IIIc		0	pT3b	< 1cm	primary	PB
#11	HGSOC	serous	hg	IIIc	pN1a	0	pT3c	< 1cm	primary	PB / MA
#12	HGSOC	serous	hg	IIIc	pN0	0	pT3c	macroscopic tumor free	primary	PB / MA
#13	HGSOC	serous	hg	IV	ypN1a	1	ypT3c	<1 cm	primary	PB
#14	HGSOC	serous	hg	IIIc	pN0	0	pT3c	> 1cm	primary	PB
#15	HGSOC	serous	hg	IIIc	pN0	0	pT3c	macroscopic tumor free	primary	PB / MA *
#16	HGSOC	serous	hg	IIIc	Nx	0	pT3c	macroscopic tumor free	recurrent	PB / MA
#17	HGSOC	serous	hg	IVb	pNX	1	pT3c	< 1cm	primary	PB /MA

#18	Uterin Sarcoma		hg			0	pT3b		primary	MA
#19	Borderline Tumor				pN0		pT3b			MA
#20	HGSOC	serous	hg	IIIc	ypN1b	0	pT3c	macroscopic tumor free	primary	PB /MA
#21	MMMT		hg		pN1a	0	pT3c	< 1cm	primary	PB /MA
#22	HGSOC	serous	hg	IIIc	pNmi	0	pT3c	macroscopic tumor free	recurrent	PB /MA
#23	HGSOC	serous	hg	IIIc	ypN1b	0	pT3c	<1 cm	primary	PB
#24	HGSOC	serous	hg	IIIc		0	pT3c	> 1cm	primary	PB
#25	HGSOC	serous	hg	IIIc	pN1a	0	pT3c	macroscopic tumor free	primary	PB / MA *
#26	HGSOC	serous	hg	IV	pN0	1	pT3c	< 1cm	primary	PB
#27	HGSOC	serous	hg	IIIc	pN0	0	pT3c	1 cm	primary	PB / MA
#28	HGSOC	serous	hg	IIIc	pN0	0	pT3c	> 1cm	primary	PB
#29	HGSOC	serous	hg	IIIc	pN1a	1	pT3c	macroscopic tumor free	primary	PB / MA *

MMMT: Malignant mixed mullerian tumor
hg: high grade
lg: low grade
PB: peripheral blood
MA: malignant ascites
PB/MA*: matched samples

Supplementary table 1 includes the clinical information of 29 patients who donated peripheral blood and/or malignant ascites for the study. Parameters collected include diagnosis, grading, FIGO status, nodal involvement metastases, pT status, tumor status post surgery (residual tumor) and time of analysis (primary/recurrent).

Supplementary Table 2. UKE RNAseq cohort (n=192) used for analyses of RNA data.

		n	%
FIGO	I	3	1,6
	II	5	2,6
	III	141	73,4
	IV	38	19,8
	unknown	5	2,6
Grading	G1	1	0,5
	G2	21	10,9
	G3/G4/hg	168	87,5
	unknown	2	1,0
Stage	pT1-pT2	12	6,3
	pT3a-pT3b	16	8,3
	pT3c	160	83,3
	unknown	4	2,1
pN	N0	38	19,8
	N1	114	59,4
	Nx	40	20,8
Histological Type	serous	177	92,2
	endometrioid	1	0,5
	clear cell	2	1,0
	mucinous	1	0,5
	undifferentiated	3	1,6
	Mixed Müllerian Tumor	7	3,6
	others/ unknown	1	0,5
Tumor rest after surgery	not macroscopically visible	117	60,9
	$\leq 1cm^3$	42	21,9
	$\geq 1cm^3$	31	16,1
	unknown	2	1,0
Treatment	Carboplatin	116	60,4
	Carboplatin/ Bevacizumab	68	35,4
	no Therapy	6	3,1
	unkown	2	1,0
Age at diagnosis (y)	mean (median)	60 y (61.0y)	
Follow-up	mean (median)	43 m (39 m)	
Recurrence-free interval	mean (months)	28 m (20 m)	

Supplementary table 2 contains the clinical information of 192 patients with ovarian diagnosed and treated at the UKE. Parameters include FIGO, grading, Stage, pN, histological type, tumor rest after surgery, treatment, age, follow-up and recurrence-free interval.

Supplementary Table 3. TCGA cohort (n=517) used for analyses of RNA data.

		n	%
Race	white	458	(88.6)
	black	23	(4.4)
	asian	15	(2.9)
	others	3	(0.6)
	n.a.	18	(3.5)
Stage	Ia-Ic	15	(2.9)
	IIa-IIc	25	(4.8)
	IIIa	7	(1.4)
	IIIb	22	(4.3)
	IIIc	364	(70.4)
	IV	80	(15.5)
	n.a.	4	(0.8)
Grading	G1	5	(1.0)
	G2	64	(12.4)
	G3/G4	437	(84.5)
	GX	8	(1.5)
	n.a.	3	(0.6)
Residual disease	no macroscopic disease	102	(19.7)
	1-10 mm	233	(45.1)
	11-20 mm	32	(6.2)
	> 20 mm	92	(17.8)
	n.a.	58	(11.2)
Vascular invasion	yes	78	(15.1)
	no	68	(13.2)
	n.a.	371	(71.8)
Lymphovascular invasion	yes	120	(23.2)
	no	75	(14.5)
	n.a.	322	(62.3)
Pharmaceutical adjuvant Tx	yes	475	(91.9)
	no	26	(5.0)
	n.a.	16	(3.1)
Treatment outcome first course	complete remission	300	(58.0)
	partial remission	59	(11.4)
	stable disease	26	(5.0)
	progressive disease	37	(7.2)
	n.a.	95	(18.4)
Vital status	alive	195	(37.7)
	dead	322	(62.3)
Mean age (years)		59.8	
Mean overall survival (days)		1050	

n.a.: data not available

Supplementary table 3 contains the clinical information of 517 patients with ovarian cancer included in the TCGA cohort. Parameters include race,stage, grading, residual disease, vascular and lymphovascular invasion, adjuvant chemotherapy, outcome, vital status, age and overall survival.

Supplementary Table 4. Fluorochrome-conjugated antibodies.

Antibody	Clone	Company
anti-CD3	OKT3	Biolegend
anti-CD14	M5E2	Biolegend
anti-CD16	3G8	Biolegend
anti-CD68	82A	Biolegend
anti-CD163	GHI/61	Biolegend
anti-CD86	IT2.2	Biolegend
anti-CD34	8G12	BD Bioscience
anti-EpCAM	9C4	Biolegend
anti-CD19	HIB19	Biolegend
anti-CD56	HCD56	Biolegend
anti-TIGIT	A15153G	Biolegend
anti-CD226	11A8	Biolegend
anti-HLA DR	L243	Biolegend
anti-CD39	A1	BD Bioscience
anti-CD73	AD2	Biolegend
anti-LAG-3	7H2C65	Biolegend
anti-TIM-3	F38-2E2	Biolegend
anti-CD204	7C9C20	Biolegend
anti-CD206	15-2	Biolegend
anti-PD1	EH12.2H7	Biolegend

Supplementary table 4 provides a list of stained antibodies with associated clone number, as well as the company from which the antibody was purchased.

Supplementary Table 5. Pearson correlation between mRNA levels (TCGA cohort)

CD163	C204	n.s.
	CD206	0,519**
	SIRPa	0,218**
	CD73	n.s.
	CD39	0,537**
	CD47	0,194**
C204	CD206	0,973**
	SIRPa	1,000**
	CD73	0,937**
	CD39	0,901**
	CD47	n.s.
CD206	SIRPa	0,973**
	CD73	0,917**
	CD39	0,902**
	CD47	n.s.
SIRPa	CD73	0,937**
	CD39	0,901**
	CD47	n.s.
CD73	CD39	0,857**
	CD47	n.s.
CD39	CD47	0,097*

* p< 0.05

**p>0.005

n.s. not significant

Supplementary table 5. To validate the results from the UKE RNAseq cohort, correlation analyses of the same parameters were performed in a second cohort of the TCGA cohort. The TCGA cohort contained 517 patients. Correlative analyses of the expression was performed by the pearson´s test. *p < .05, **p < .01, ***p < .001.

3 Supplementary Figures

Supplementary Figure 1.

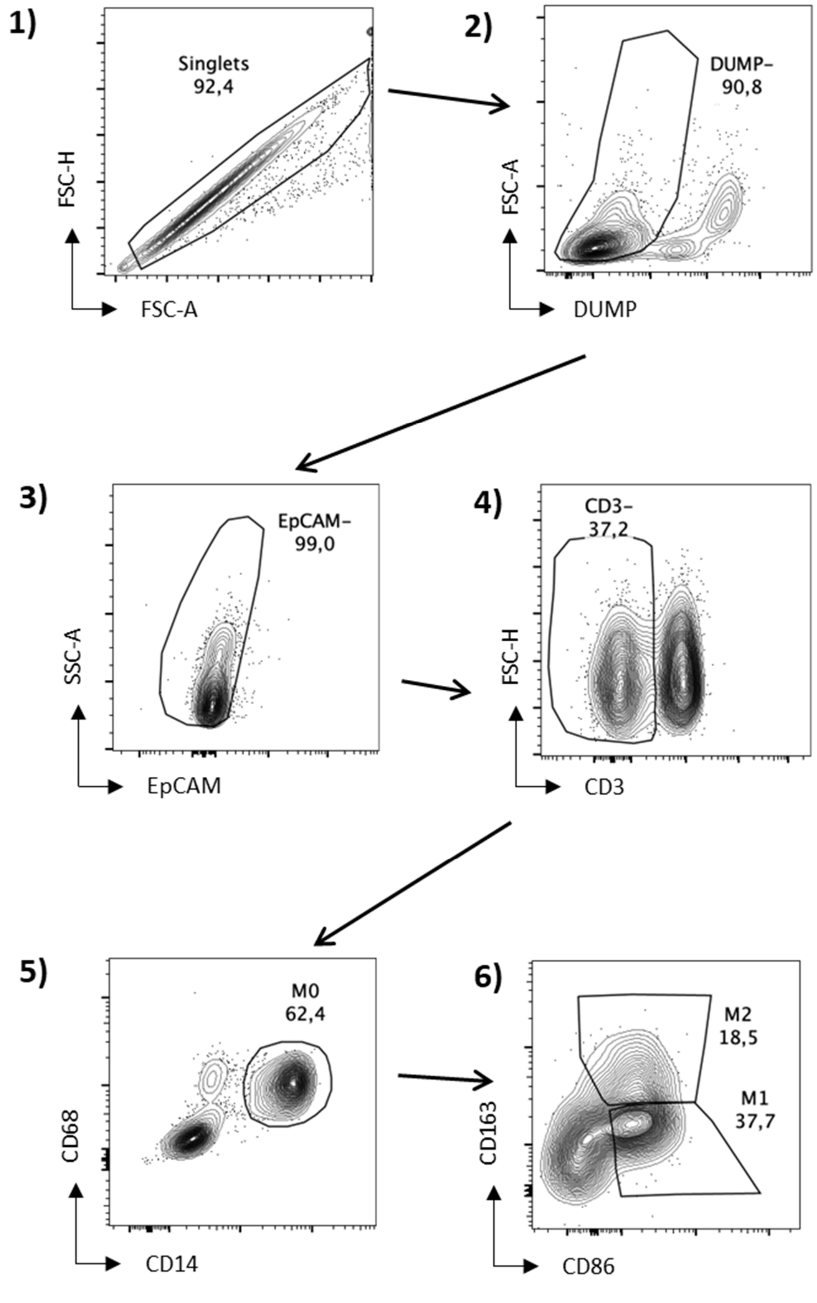

Supplementary Figure 1. Gating strategy for the phenotypic (multiparametric flow) analyses. Gating strategy used to identify $CD68^{+}CD14^{+}$ macrophages/monocyte-derived macrophages and their $CD163^{+}CD86^{+}$ M2 and $CD163^{-}CD86^{+}$ M1 subpopulations from the malignant ascites (MA) and peripheral blood (PB). The same gating strategy was used to analyze samples from PB and MA: after elimination of doublets (1) exclusion of cell debris, B cells and NK cells was performed via the *DUMP channel* (2). Next, Ovarian cancer cells were identified on the basis of their expression of EpCAM (3). T cells were defined as $CD3^{+}$ (4). $CD14^{+}CD68^{+}$ cells were identified within the remaining cells (5). Within this population, M2 and M1 subpopulations were defined on the basis of their expression of CD163 and CD86 (6).

Supplementary Figure 2.

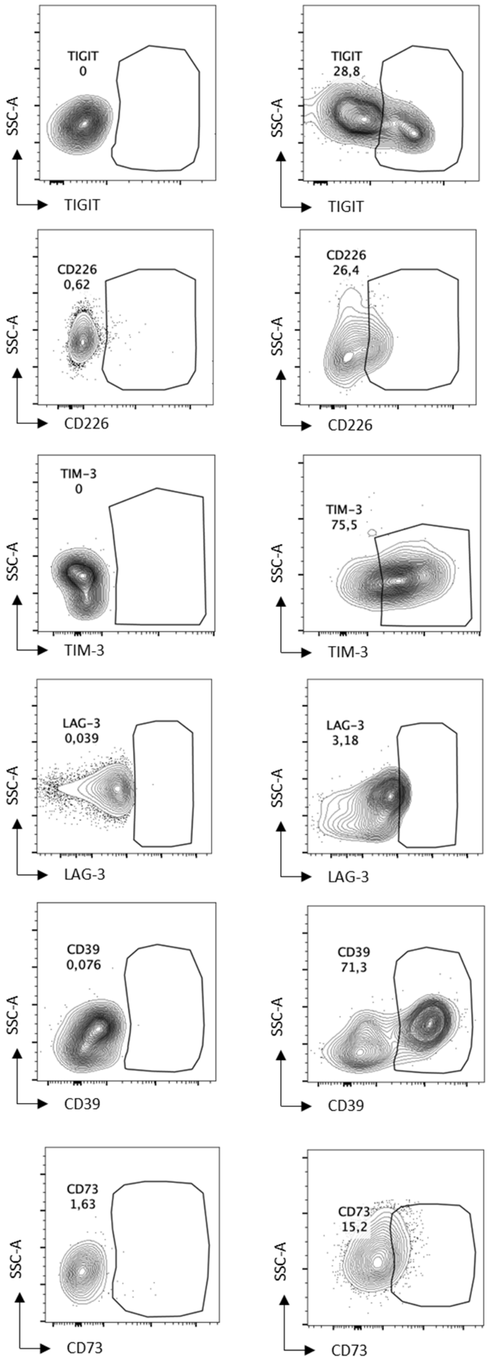

Supplementary Figure 2. Expression of regulatory receptors on CD14^{+}CD68^{+} macrophages from HGSOC patients in comparison to FMO. The surface expression of TIGIT, CD226, TIM-3, LAG-3, CD39 and CD73 was compared in malignant ascites (MA) aspirates from patients with HGSOC. The left panel represent the fluorescence minus one (FMO) control, whereas the right panel demonstrate the positive marker expression, respectively.

Supplementary Figure 3.

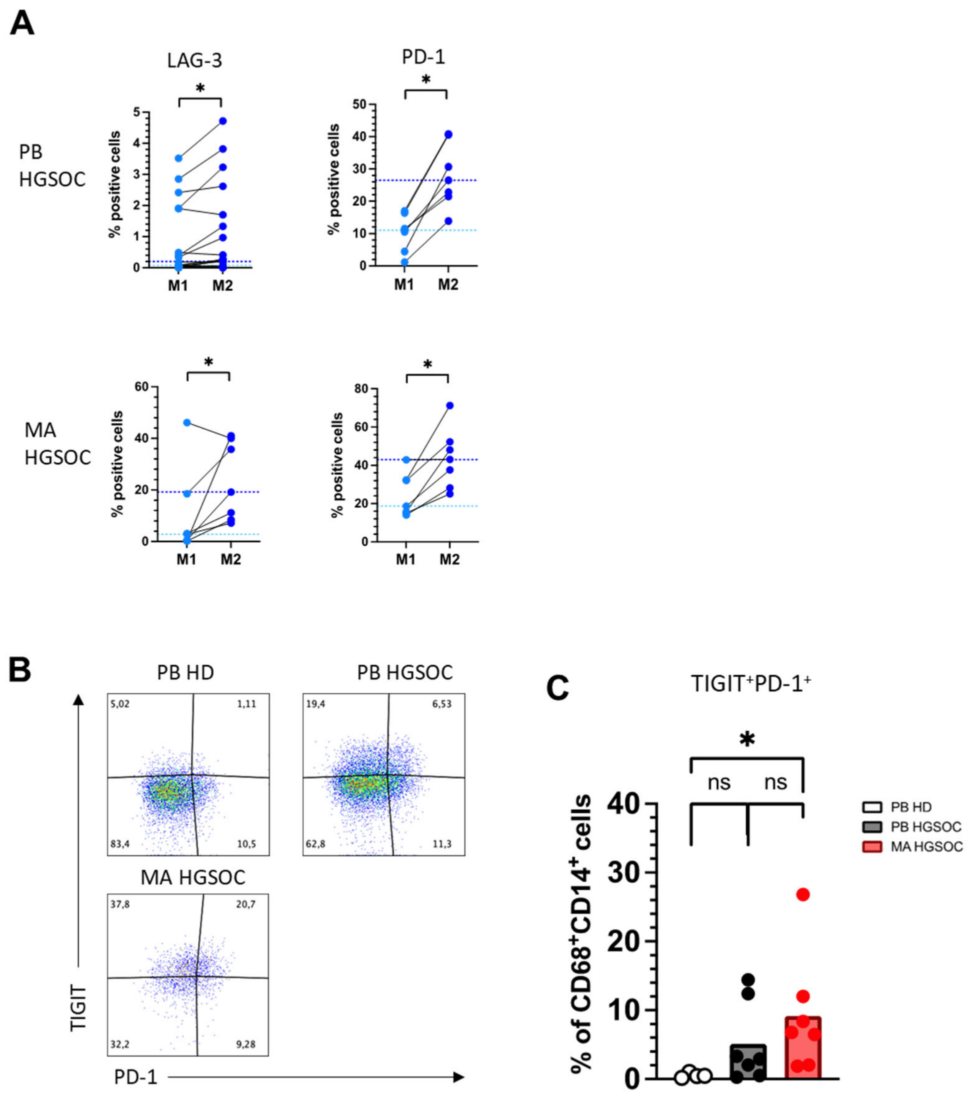

Supplementary Figure 3. Expression of LAG-3 and PD-1 on CD14$^+$CD68$^+$ macrophages from HGSOC patients vs. healthy controls. The surface expression of LAG-3 and PD-1 was compared on macrophages derived from the peripheral blood (PB) of healthy donors (HD) versus PB and malignant ascites (MA) aspirates from patients with HGSOC. (A) Summary data showing the expression of the co-regulatory receptors on CD14+CD68+ macrophages. (B) Exemplary flow plots display characteristic co-expression patterns of TIGIT and PD-1. (C) Summary data showing the frequency of macrophages co-expressing TIGIT and PD-1. P values were obtained by the ANOVA and Kruskal-Wallis test and by the Wilcoxon matched-pairs signed rank test. *P<0.05

Supplementary Figure 4.

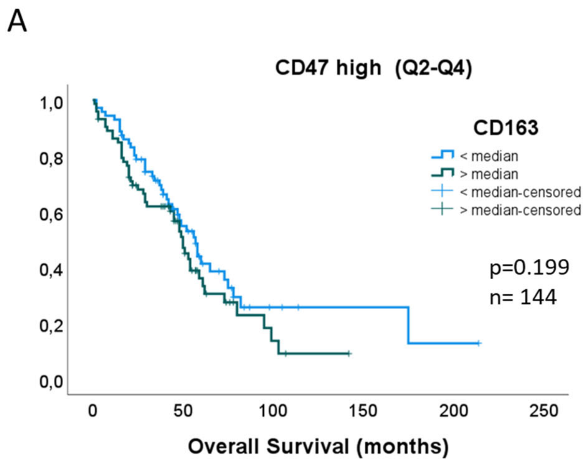

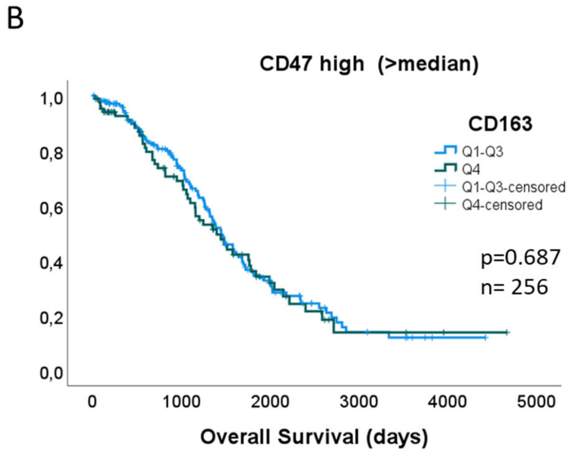

Supplementary Figure 4: High expression of CD163 is not associated with poor outcome in the CD47 high expression group. Kaplan Meier survival curves showing the overall survival of patients with high (gene expression > the median) or low (gene expression < the median) CD163 gene expression in the subgroup of CD47 high expressors within the UKE cohort (A) and the TCGA (B) cohort.

Supplementary Figure 5.

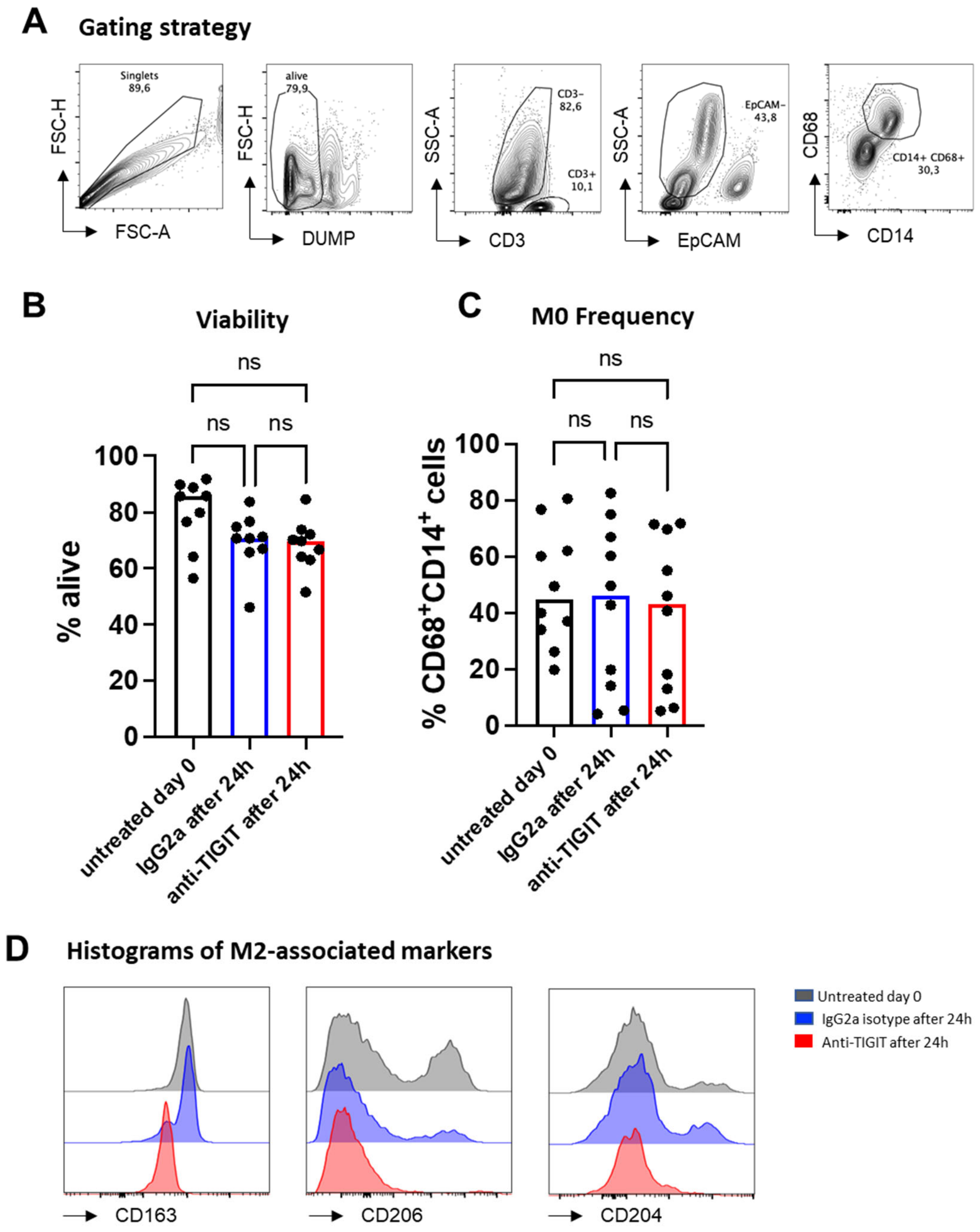

Supplementary Figure 5. Anti-TIGIT induced repolarization of primary macrophages. The multiparametric flow (MFC) gating strategy used is illustrated in **(A).** The viability of the MA-derived TAMs was assessed after 24h treatment with anti-TIGIT or respective isotype control **(B).** The percentages of $CD68^{+}CD14^{+}$ (M0) MA-derived TAMs are depicted as the median ± SD **(C)**. Proteinexpression of the M2 associated markers CD163, CD206 and CD204 on CD68+CD14+ TAMs derived from the malignant ascites (MA) are exemplary illustrated during the TIGIT blockade, respectively **(D).** P values were obtained by Anova and Friedman test.
